# Implementation of the Personalized Automated Reading Delivery System as a Component of Precision Medical Education: Prospective Crossover Study

**DOI:** 10.2196/81717

**Published:** 2026-09-21

**Authors:** William Gatenby, Sumeet R Gopwani, Glenn E Woodworth, McKenzie Hollon, Stacey Kaltman

**Affiliations:** 1 Department of Anesthesiology, MedStar Georgetown University Hospital, 3800 Reservoir Rd NW, Washington, DC, 20007, United States, 1 202-444-2000, 1 (202) 444-8854; 2 Department of Anesthesiology and Perioperative Medicine, Oregon Health and Science University, Portland, OR, United States; 3 Department of Anesthesiology, Emory University School of Medicine, Atlanta, GA, United States; 4 Department of Psychiatry, Georgetown University School of Medicine, Washington, DC, United States

**Keywords:** precision medical education, educational curricula, teaching and learning approaches and tools, technology in education, anesthesia education

## Abstract

**Background:**

Precision medical education (PME) is an emerging concept that aims to improve physician education by tailoring curricula specifically to each learner. The basic framework behind PME requires collecting data on each individual learner, identifying gaps in knowledge, and then applying direct educational interventions. However, logistical difficulties in the delivery of such interventions hinder the implementation of PME in existing academic programs.

**Objective:**

We developed the Personalized Automated Reading Delivery System (PARDS) software application to support a potential PME intervention, aiming to increase the volume of relevant scientific literature read by resident physicians. The PARDS analyzes surgical case lists for the following day, selects relevant scientific articles according to predetermined keywords, and delivers each article via email to an anesthesiology resident physician. This technology may assist in the application of personalized educational interventions, and we evaluated the feasibility of implementing the PARDS in a population of medical learners.

**Methods:**

From July 2018 to December 2018, a pilot prospective crossover study was performed with N=10 postgraduate year 2 (PGY2) anesthesiology residents in an academic medical center (response rate 10/10, 100%). Residents were randomly assigned to the PARDS intervention group for 2 of the 4 months of the study period. The primary outcomes were organizational viability (evaluated by successful delivery of selected articles by the PARDS, required faculty hours, and total expenses) and acceptability in the study population (assessed by the number of reported weekly reading hours). An analysis using a linear mixed model for repeating measures was performed to compare weekly reading hours between intervention and nonintervention conditions.

**Results:**

Total reported faculty time involvement for the study period was 24 hours. The cost estimate for implementation ranged from US $2360 to US $8360. The mean difference of recorded reading hours by residents while using the PARDS vs not using the PARDS for months 1, 2, 3, and 4 of the study was 2.56 (95% CI −0.03 to 5.15; *P*=.053), 0.44 (95% CI −2.15 to 3.03; *P*=.74), 0.99 (95% CI −1.38 to 3.36; *P*=.41), and 3.7 (95% CI 1.34 to 6.08; *P*=.002), respectively.

**Conclusions:**

The PARDS was successfully implemented in an academic medical center. Calculated costs indicate that the PARDS is an inexpensive intervention compared to contemporary alternatives. The effect on resident reading requires further research.

## Introduction

Physicians in medical residency programs value educational content tailored to their professional goals, but modern curricula often fail to meet this standard [1-5]. To address this discrepancy, medical educators have proposed incorporating concepts from precision medical education (PME) [6,7]. PME is an emerging framework in medicine that provides personalized education to residents by (1) gathering data on the learner, (2) identifying gaps in knowledge, and (3) intervening through the direct delivery of resources [6,7]. With the proliferation of data collection tools (eg, web-based dashboards for learner assignments and lecture content, electronic health records [EHRs]) and the advent of AI, the potential to truly tailor medical residency education to each learner has reached unprecedented heights.

To this end, various aspects of PME models in residency programs are currently undergoing investigation. Schaye et al [8] successfully implemented a software program in an internal medicine residency where clinical documentation was collected and rated on quality, indicating areas for targeted intervention. Laufer et al [9] proposed a “future model” for radiology training that uses AI to survey patient and resident clinical experiences and match specific pathologies with less-experienced residents.

Despite this progress, significant barriers to the implementation of PME exist. For instance, while potentially useful, AI also remains largely untested in this space, and the best way to use it without furthering inequity or bias has yet to be explored [10]. Simply possessing large amounts of data on learners does not ensure insightful analysis of those data or a consequential change in a medical learner’s education [10,11]. The most commonly cited barriers to PME in residency education remain the monetary and faculty time investments required to assess residents and deliver targeted resources at the individual level [6-9].

We have identified a potential opportunity for a PME intervention in the delivery of educational materials to anesthesiology resident physicians, with the goal of increasing their reading of curated educational content. Past studies have demonstrated that providing resident physicians with specific educational resources resulted in positive outcomes in both examination scores and learner satisfaction [12,13]. However, the time and expense required to analyze the operating room schedule, match appropriate educational content, and supply residents with curated, evidence-based articles have prevented the implementation of such a PME system.

To address these concerns, we developed the Personalized Automated Reading Delivery System (PARDS) as a component of a PME intervention. The PARDS is a software program that supplies anesthesiology residents with curated articles matched to surgical cases in which they will participate on the subsequent day. The goals of this study are to report on the feasibility of implementing the PARDS in an academic medical center and its impact on participating residents as defined by changes in reading behavior. This work is relevant to medical educators who are endeavoring to create efficient and personalized perioperative training through the use of technology and offers a valuable proof of concept for the design, implementation, and expansion of identical or similar PME tools.

## Methods

### Setting

To examine the impact and feasibility of using the PARDS software application in an academic medical center, a pilot, prospective, randomized crossover study was conducted in 2018. Participants comprised postgraduate year 2 (PGY2) anesthesiology residents at MedStar Georgetown University Hospital (N=10, response rate 10/10, 100%). All PGY2 anesthesiology residents at this institution participated in the study with no exclusions.

### Intervention and Study Design

**Figure 1.**
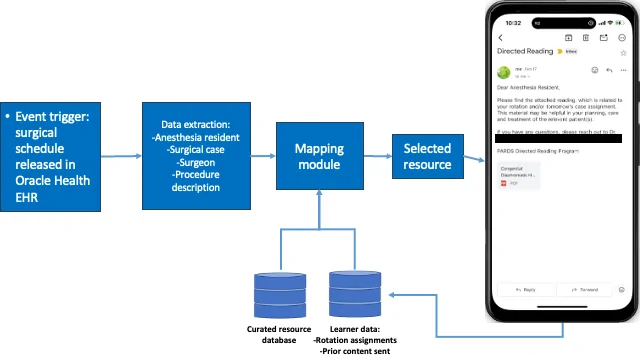
Mechanism of the Personalized Automated Reading Delivery System (PARDS). The PARDS automatically monitors the surgical schedule in the Oracle Health electronic health record ([EHR], event trigger) and extracts key pieces of data (data extraction). When extracted data match predetermined keywords associated with content in the curated resource database (mapping module), the content is selected and delivered via email.

The PARDS software application was designed to automatically query resident operating room case assignments and deliver a personalized evidence-based article from the curated resource database each evening (Figure 1). When extracted data matched predetermined keywords associated with an article in the curated resource database, the article was selected. Case assignments were obtained from the Oracle Health (formerly Cerner) EHR MPages reporting tool (Oracle Corporation), accessed via the MPages web service. The output data were processed through a JSON conversion, removing protected health information and storing the remaining data (resident name, primary attending surgeon name, surgical case name, and surgical comments) in a cloud database. Resident rotation schedules were manually preprogrammed into the database. Each resident’s daily case assignment and the current rotation were obtained from the database using a custom algorithm developed with the React Native framework (version 0.51; Meta Platforms Inc), a JavaScript framework, on the open-source Expo platform (version 24.0.0; Expo Technologies Inc). The React Native algorithm used a keyword mapping module to link surgical case names, surgical comments, and the name of the primary attending surgeon to the curated list of educational content using natural language processing. A notification function in the React Native application emailed the matched article to participants at approximately 5 PM for relevant surgical cases the following day. Residents received a maximum of 12 articles per rotation. Articles selected and emailed to the residents were cataloged by the PARDS after selection in order to avoid it sending duplicate articles.

The curated resource database was created by anesthesiology department faculty, who were asked to select 12 evidence-based articles related to an anesthesia rotation for each of the 12 anesthesia rotations offered to residents for a total of 144 articles. Articles were assessed for and assigned keywords related to learning objectives for each rotation. Faculty then reported the number of hours spent selecting resources for the database.

**Figure 2.**
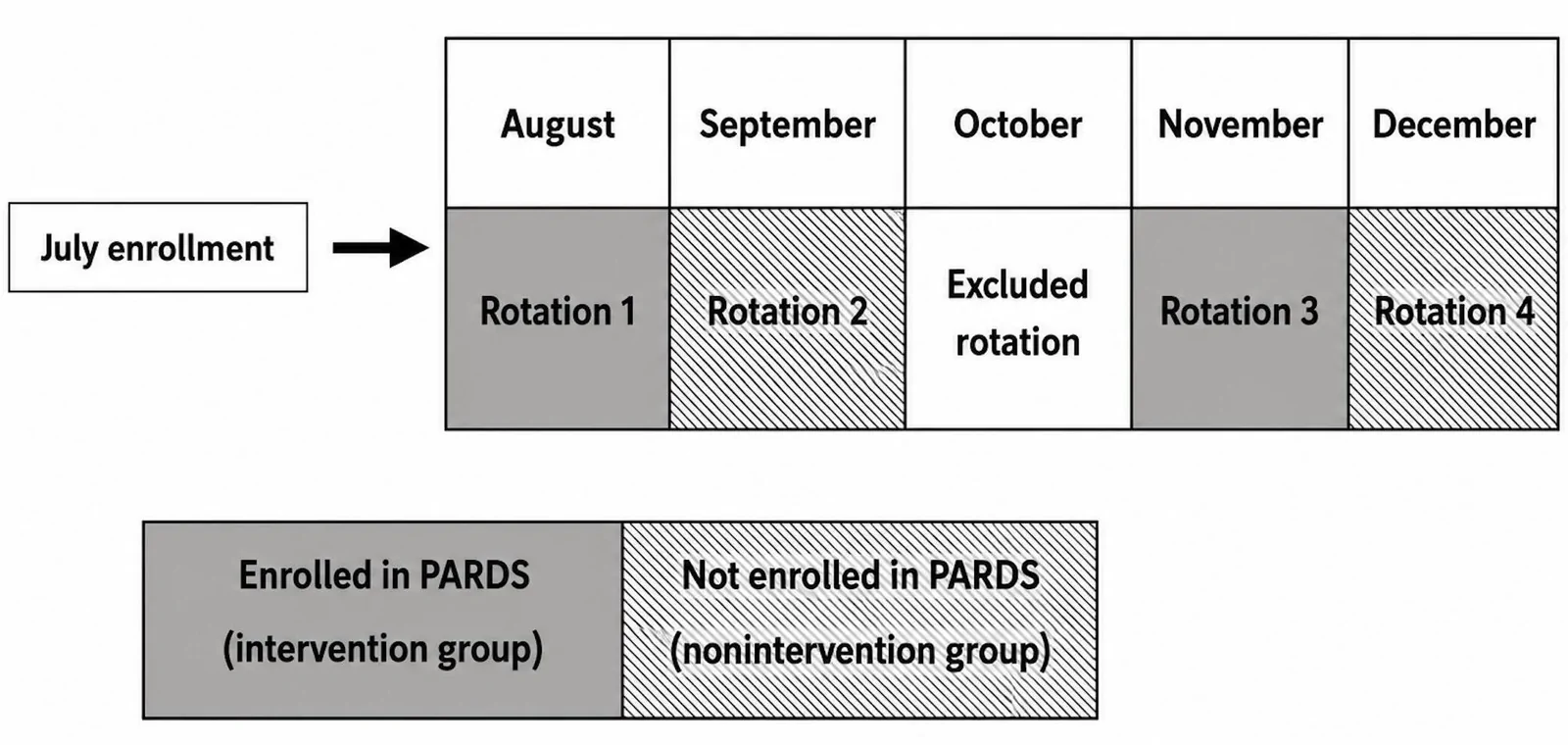
Example of study progression for a participating postgraduate year 2 resident physician in the Personalized Automated Reading Delivery System (PARDS). Resident physicians were recruited in July 2018 and participated in 4 included anesthesia rotations and 1 excluded medicine rotation over a 5-month period. For each rotation, resident physicians were either enrolled in the PARDS (intervention group) or not enrolled (nonintervention group).

During the study period, participating residents engaged in 4 anesthesiology rotations as well as 1 medicine rotation, which was excluded from this study. All rotations were 1 month in duration. Residents received articles via the PARDS during 2 rotations throughout the study period, and during the remaining 2 rotations they did not receive articles (Figure 2). For each rotation, residents who received articles comprised the intervention group, and those who did not formed the nonintervention group. Residents were randomized to the intervention or nonintervention group during their rotations using a balanced randomization approach. Randomization was performed with a random number generator and controlled for equal balancing among rotations, residents, and time points as best possible.

All residents regardless of enrollment in the PARDS possessed electronic access to the database of curated articles for each rotation, and while they were encouraged to use the database, those in the nonintervention group did not receive specific instruction and were not sent articles.

### Outcomes

The number of reported reading hours was used to assess the impact of the PARDS intervention. As the goal of the PARDS is to increase specific educational resource use, a change in the number of hours spent reading resources related to clinical practice by each resident while participating in the PARDS intervention was viewed as a proxy for the effect on the study population.

In accordance with prior literature [12], resident reading hours were measured with survey data. Paper surveys were administered by a research assistant at the end of each month, which contained a single question: “On average, how many hours did you spend reading materials relevant to your clinical practice per week, over the prior month?”

Feasibility of the intervention was evaluated both by successful implementation of the PARDS and by the accrued faculty hours and expenses required to implement the system. Successful implementation was defined as the PARDS assessing the surgical case list for the following day, assigning a specific learning resource/article, and emailing the assigned reading to each participating resident prior to the scheduled surgical procedure.

### Analysis of the Outcomes

The difference in mean resident reading hours between the intervention and nonintervention groups was analyzed using a linear mixed model for repeated measures for the 4 months that residents participated in this study. Analyses were conducted in Stata 18 (StataCorp LLC). The threshold for statistical significance was α=.05 for all comparisons.

The steps needed to develop and implement the PARDS were recorded by the study team, and the associated expenses were calculated. Faculty and programmer compensation was determined based on standard market rates for anesthesiologists in the mid-Atlantic region in 2018 (the year the study was conducted).

### Ethical Considerations

This study was conducted with the approval of the institutional review board of Georgetown-MedStar (ID 2018‐0515). All participants provided written informed consent. Study participants were not compensated for participation in the study.

## Results

In evaluating feasibility, we found that several steps were required to initiate the PARDS (summarized in Table 1). Anesthesiology department faculty members reported spending 2 hours per rotation selecting educational materials, for a total of 24 hours across 12 rotations. An Oracle Health and MPage developer required approximately 20 hours to build the surgical assignment data report. Development of the React Native application, including the back-end database, required 30 hours for a junior programmer. The initial expense required for PARDS development was calculated at US $8360, with a US $10 per month recurring expense. The initial expense could be as low as US $2360 depending on faculty involvement. Following this stage, the implementation of the PARDS was successful, as each resident in the intervention group received at least 1 article in reference to the surgical case listed for the following day.

**Table 1.** Calculated expenses for implementation of the Personalized Automated Reading Delivery System.

Expense category	Monetary expenses (US $)	Academic faculty time (hours)	Programmer time (hours)	Notes
Reading development	6000 (onetime) or 0 (unfunded, 2 hours/subspecialty faculty rotation mentor)	24	0	Rotation mentor faculty identify 12 articles: 12 rotations × 2 hours = 24 hours × US $250/h = US $6000; or, invested core faculty experts spend 2 hours to identify 12 readings to distribute
EHR^a^ extraction	860 (onetime)	0	20	MPage developer (or Epic Reporting programmer): 20 hours × US $43/hour = US $860
React Native and database development	1500 (onetime)	0	30	Back-end developer: 30 hours × US $50/hour = US $1500
Materials	<10/month (continuing)	0	0	Open-source Expo platform using React Native: free; or, cloud database <US $10/month
Annual database update	0 (unfunded, 4 hours for a program administrator)	4	0	Residency coordinator updates resident participants and rotation schedule annually
Total	2360-8360	28	50	—^b^

a EHR: electronic health record.

b not applicable.

In month 1 of the study, anesthesiology residents in the intervention group reported reading a mean of 2.56 more hours (95% CI −0.03 to 5.15; *P*=.053) than the nonintervention group (Table 2). In months 2 and 3, anesthesiology residents in the intervention group reported reading a mean of 0.44 more hours (95% CI −2.15 to 3.03; *P*=.74) and 0.99 more hours (95% CI −1.38 to 3.36; *P*=.41) than the nonintervention group, respectively. In month 4 of the study, anesthesiology residents in the intervention group reported reading a mean of 3.7 more hours (95% CI 1.34 to 6.08; *P*=.002) than the nonintervention group. This difference in reading hours between the intervention and nonintervention groups was statistically significant in month 4. The actual reported reading hour data are available in Multimedia Appendix 1.

**Table 2.** Estimated mean difference of recorded reading hours by resident physicians when participating in the Personalized Automated Reading Delivery System ([PARDS], intervention) and when not participating in the PARDS (control) for each month of the study.

Month of study	Mean difference in reading time between intervention group and control group (hours)	95% CI	*P* value
Month 1	2.56	−0.03 to 5.15	.053
Month 2	0.44	−2.15 to 3.03	.74
Month 3	0.99	−1.38 to 3.36	.41
Month 4	3.7	1.34 to 6.08	.002

## Discussion

### Principal Findings

This study sought to evaluate both the feasibility and the impact on reading hours of the PARDS in a medical education environment. The PARDS was successfully implemented with minimal cost and faculty hours in the department of anesthesiology of an academic medical center. As intended, residents received relevant selected readings (a maximum of 12 per rotation) the evening prior to a surgical procedure while participating in the PARDS intervention rotation. The majority of time investment (software programming, curated resource database article selection) was spent on initial implementation, with minimal maintenance required, and monetary expenses were nominal when compared to contemporary alternatives for curricular interventions in anesthesia residency programs (Table 1). For example, the popular online educational resources Anesthesia Toolbox and TrueLearn typically cost programs US $100/resident/year and approximately US $400‐800/resident/year, respectively [14,15]. Hands-on curricular interventions in residencies (simulation labs, in-person trainings) require even greater investments, with reported costs of more than $10,000 for onetime implementation for some examples [16-18]. By our estimate, the PARDS could be initiated for as little as $2360 with a small recurring cost per year, offering an affordable educational intervention. This cost estimate could be even further reduced with the use of AI services to develop the curated article collection, as faculty hours and the associated cost constitute a large part of the projected expenses.

Use of this technology by the study population was promising. During the first month of the 4-month study period, participants showed a considerable but not statistically significant increase in reading hours. Importantly, there was a significant increase in reading hours during month 4. The lack of statistical significance in the first 3 months of the study period may be due to a failure of the PARDS to impact the resident physician population. However, the increased use of the PARDS during month 4 of the study period could be explained by increased familiarity with the system by resident physicians over time. Overall, these findings suggest that the PARDS can potentially impact resident reading hours. Aligned with this, several studies have shown that residents respond positively to directed reading interventions. Journal clubs where residents are provided educational readings have long been correlated with increases in reading hours [19,20], and Watkins et al found that general surgery residents used educational resources when sent weekly emails [21], similar in function to the PARDS.

Based on the above evidence, it is our belief that the PARDS could function as a component of a PME intervention. The most current accepted aim of PME is the efficient delivery of the right educational intervention to the right learner at the right time [8], and as a system for distributing educational materials at specific time points, the PARDS could assist residency programs in intervening in gaps in knowledge. As a component of a residency curriculum, the PARDS is an attractive tool, providing guided subjects on which to design educational discussions as both attending physicians and residents may be sent the same curated article.

Notably, this software program is transferable to other institutions. Little adaptation would be required by institutions who use Oracle Health EHR, from which the current PARDS extracts surgical case information. Because multiple medical specialties train residents through participation in surgical cases, the PARDS could be applied in a variety of residency programs. Finally, as mentioned previously, AI services may further reduce both cost and required faculty input, lowering barriers for the implementation of this technology.

### Limitations

It is important to note several limitations of this pilot study. The sample population consisted of 10 residents, all from a single institution and residency program, reducing statistical power and generalizability. The amount that a resident reads during any given time period can be affected by a myriad of confounders, which would be difficult to control for. Data on reading were self-reported, with residents recording average weekly reading hours once per month, further increasing the risk of reporting biases in participants. Residents participated in month-long rotations but estimated weekly reading hours, for which the last week of reporting may differ by approximately 1 day per rotation.

Finally, this study did not distinguish sources of reading materials, meaning that reported reading volume could consist of resources not provided by the PARDS.

### Next Steps

Further research on the utility of the PARDS is therefore warranted. Important next steps include evaluating the effect of the PARDS in a larger participant pool as well as assessing knowledge acquisition and impact on examination scores in residency cohorts. To this end, the PARDS software is currently being incorporated into a multi-institutional PME project developed by The Anesthesia Research Group on Education Technology (TARGET). We believe that evaluating the feasibility and acceptance in this larger study population will shed light on the potential utility of this developing technology.

### Conclusions

This pilot study demonstrates the successful implementation of the PARDS in an academic medical center, which may function to increase reading hours among anesthesia residents. We are unaware of any other innovations to automate the process of matching evidence-based articles to specific resident clinical experiences in real time, and we believe that these efforts can contribute to the growing efforts to leverage technology to evolve and improve medical education.

## Supplementary material

10.2196/81717 Multimedia Appendix 1 Reported reading hours by postgraduate year 2 anesthesiology resident physicians per week during study period.

## Abbreviations

EHR: electronic health record
PARDS: Personalized Automated Reading Delivery System
PGY2: postgraduate year 2
PME: precision medical education
TARGET: The Anesthesia Research Group on Education Technology
