# Supplementary material for: Implementation of the Personalized Automated Reading Delivery System as a Component of Precision Medical Education: Prospective Crossover Study
Source: JMIR Form Res. 2026 Sep 21;10:e81717. doi: 10.2196/81717 (PMC13592747; doi:10.2196/81717)
Supplement: Supplementary file 1 [file formative-v10-e81717-s001.docx]

| Anesthesiology Resident Physician | Month 1 | Month 2 | Month 3 | Month 4 |
| --- | --- | --- | --- | --- |
| 1 | 6 | 2 | 2 | 3 |
| 2 | 4 | 4 | 4 | 4 |
| 3 | 9 | 2 | 6 | 2 |
| 4 | 12 | 9 | 8 | 5 |
| 5 | 10 | 7 | 8 | 8 |
| 6 | 6 | 3 | 7 | 4 |
| 7 | 9 | 6 | 4 | 12 |
| 8 | 3 | 2 | 2.5 | 4 |
| 9 | 12 | 5 | 12 | 15 |
| 10 | 10 | 10 | 10 | 10 |

Reported reading hours by postgraduate year 2 (PGY2) anesthesiology resident physicians per week during study period.

Gray = enrolled in the Personalized Automated Directed Reading Program.
White = Not enrolled in the Personalized Automated Directed Reading Program.
